# Head rotation improves airway obstruction, especially in patients with less severe obstructive sleep apnea without oropharyngeal collapse

**DOI:** 10.1371/journal.pone.0268455

**Published:** 2022-05-24

**Authors:** Shi Nee Tan, Jong-Min Kim, Jisun Kim, Chung Man Sung, Hong Chan Kim, Jongho Lee, Sang Chul Lim, David P. White, Hyung Chae Yang, D. Andrew Wellman

**Affiliations:** 1 Department of Otolaryngology-Head and Neck Surgery, Chonnam National University Medical School and Chonnam National University Hospital, Gwangju, Republic of Korea; 2 School of Medicine, KPJ University College, Nilai, Negeri Sembilan, Malaysia; 3 School of Mechanical Engineering, Gwangju Institute of Science and Technology (GIST), Gwangju, Republic of Korea; 4 Division of Sleep and Circadian Disorders, Department of Medicine and Neurology, Brigham and Women’s Hospital and Harvard Medical School, Boston, MA, United States of America; AUSL della Romagna, ITALY

## Abstract

**Purpose:**

Head rotation is thought to have an effect on obstructive sleep apnea (OSA) severity. However, keeping the head rotated fully during sleep is difficult to maintain, and the effect of head rotation is not the same in all OSA patients. Thus, this study aimed to identify whether less head rotation has an effect on airway patency and determine the responder characteristics to the head rotation maneuver (HRM).

**Methods:**

We recruited 221 patients who underwent overnight polysomnography and drug-induced sleep endoscopy (DISE) in a tertiary hospital from June 2019 to July 2020. Airway patency and the site of airway collapse were determined in the supine position with the head at 0, 30, and 60 degrees of rotation (HRM0°, HRM30°, and HRM60°, respectively) during DISE. The site of collapse was determined using the VOTE classification system: the velum (palate), oropharyngeal lateral walls, tongue base, and epiglottis. Each structure was labeled as 0, 1, or 2 (patent, partially obstructed, and completely obstructed, respectively). Airway response to the HRM30° and 60° and the clinical characteristics associated with airway opening were analyzed.

**Results:**

The study population had a median age of 52 (25–61) years, a body mass index of 26.7(24.6–29.4) kg/m^2^, and the apnea-hypopnea index (AHI) of 28.2(13.7–71.9) events/h. HRM influenced airway patency positively not only with HRM60° (p<0.001) but also following limited rotation (HRM30°, *p*<0.001). Patients with tongue base (40.0% with HRM 60°) and epiglottic (52.6% with HRM 60°) collapse responded particularly well to HRM. Multivariate analysis revealed that lower AHI (*p*<0.001) and an absence of oropharyngeal lateral walls collapse (*p* = 0.011) were significant predictors of responders to HRM.

**Conclusion:**

Head rotation improved airway obstruction in OSA patients, even with a small degree of rotation, and should be further explored as a potential form of therapy in appropriately selected patients.

## Introduction

Treatment of obstructive sleep apnea (OSA) is challenging. Thus, several treatment methods have been explored, including positional therapy [1–6]. Positional therapy involves turning the head and trunk to a lateral position. However, this may be uncomfortable during sleep and is not easily maintained. On the other hand, head rotation, which is also considered a type of positional therapy, may be more comfortable and potentially easier to maintain during sleep. Previous studies of head rotation showed that it mimicked the airway status changes produced by moving the head and trunk to the lateral position [7, 8].

Indeed, head rotation appears to influence the severity of OSA positively. Van Kesteren et al.’s study involving 199 patients found that the apnea-hypopnea index (AHI) in the HRM0° position averaged 42.7 ± 27.1 events/h. However, when the head was rotated, the AHI was reduced to 37.3 ± 23.9 events/h [9]. Tate et al. also observed that the AHI in 11 patients was improved by 11.0 events/h (95%CI:-21.6~-0.3) with head rotation [10]. Safiruddin et al. simulated head rotation during drug induced sleep endoscopy (DISE) in two studies with 100 and 60 patients, respectively [11, 12]. They reported that head rotation decreased the frequency of airway collapse, and the airway status during the head rotation maneuver mimicked moving the head and trunk to the lateral position [7, 8].

However, to date, there is no information in these previous studies on how much the head should be rotated, although there are methods to monitor head position. HRM was treated as a dichotomous variable in previous studies. Kesteren et al. and Tate et al.’s observational studies set a threshold for head rotation at 45° [9, 10]. Walsh et al. included head rotation positions from 32° to 55° [13]. Safirrudin et al. and Vonk et al.’s simulational study also used a dichotomous classification of head rotation, and the degree of rotation was not specified [11, 12, 14]. However, we can infer from these studies that the head can be comfortably rotated up to 60° in natural sleep. Tate reported the head was rotated from up to 60° in some patients, and Walsh et al. also reported a threshold angle up to 55° during natural sleep in their study. Thus, we can assume the range of head rotation is up to 60° in natural sleep. However, these studies found that maintaining the maximal rotation during sleep was challenging. If relatively limited head rotation successfully alleviated airway collapse, it would be easier to maintain during sleep.

Another problem with head rotation is that its effect on airway patency is variable. Walsh et el. reported that pharyngeal critical pressure improved with head rotation in five subjects but worsened in another five [13]. Kerenstren et al. reported that, overall, the AHI slightly decreased from 42.7 ± 27.1 to 37.3 ± 23.9, but it remained unchanged or worsened in a considerable number of patients [9]. Furthermore, Safirrudin et al.’s DISE study reported that patients with oropharyngeal lateral walls collapse did not respond well to head rotation [11]. In addition, positional therapy, which is thought to produce similar anatomic changes as head rotation, is not recommended for everyone by the European Respiratory Society [15]. Thus, it is likely that head rotation is effective in selected patients.

If head rotation is to be considered as a potential therapeutic method, it is important to know who can benefit from this maneuver and how much the head should be rotated. Thus, we produced head rotation during sleep, identified its influence on patients with OSA according to the degree of head rotation, and analyzed the characteristics of the patients who had a good response to head rotation. This information could help motivate the development of sleep gadgets to maintain head rotation during sleep.

## Materials and methods

### Study subjects

**Fig 1:**
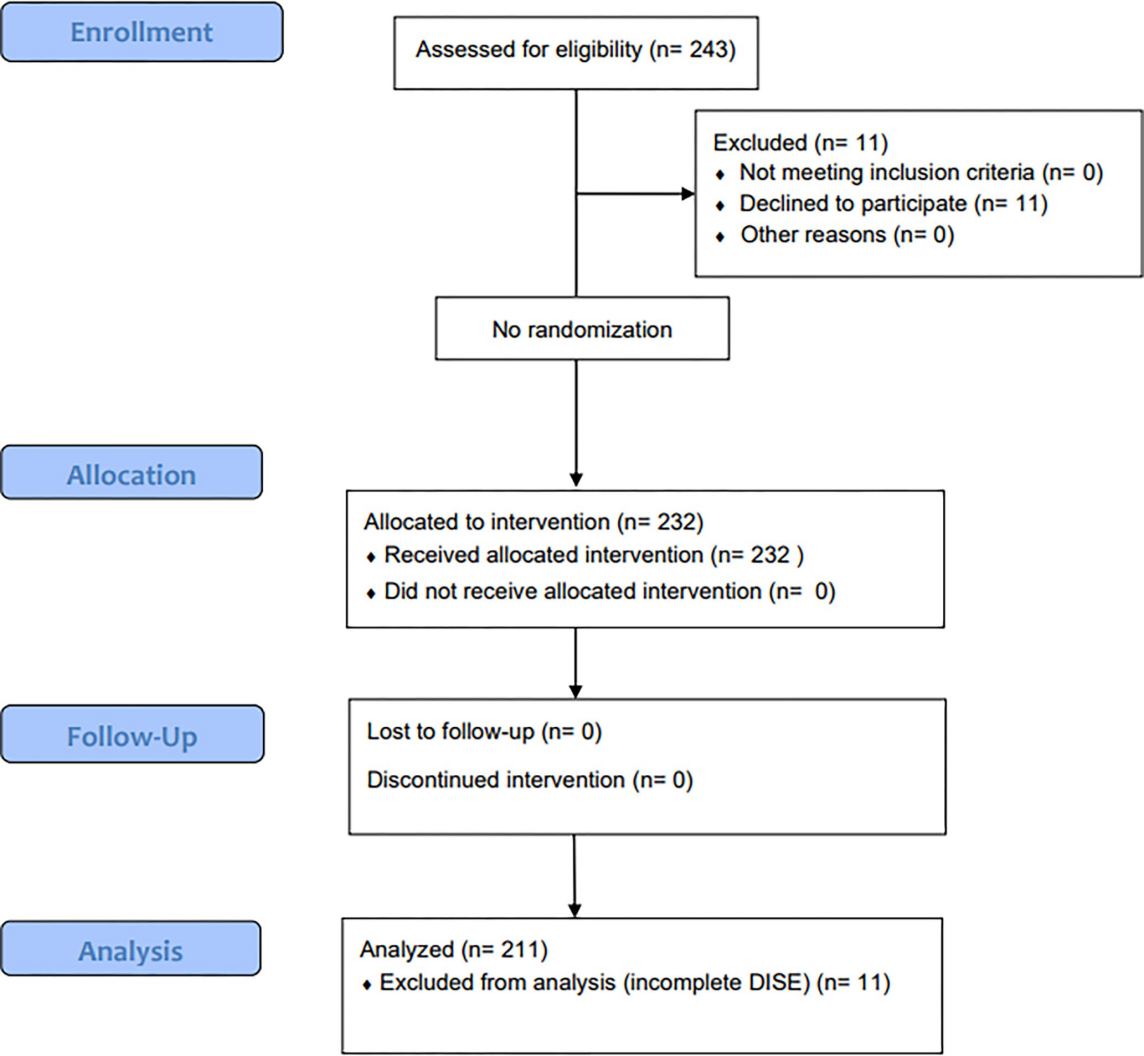
Flow chart of patient deposition. In total, 243 patients underwent initial screening for the study, and 221 patients underwent DISE with head rotation maneuvers. Patients were divided into the positional sleep apnea (POSA, n = 91) group (supine sleep position for ≥ 30 min) and the non-positional sleep apnea (non-POSA, n = 76) group (non-supine sleep position for ≥ 30 min). Patients who slept only in the supine position or only in the lateral position ≤ 30 min were excluded from the subgroup analysis (n = 54).

This study analyzed 243 consecutive patients visiting a tertiary hospital with a complaint of snoring or sleep apnea symptoms between June 2019 and July 2020. Patients who underwent both polysomnography (PSG) and DISE with HRMs were included in the analysis. In contrast, patients with incomplete PSG data, DISE data, or a history of upper airway surgery were excluded from the final analysis (Fig 1). Patients with poorly controlled systemic health conditions, pregnancy, neck injuries or head/neck complaints, or a known history of drug allergies (such as propofol allergy) were excluded from the DISE examination before study enrollment. This study was performed in accordance with the 1964 Declaration of Helsinki. The Chonnam National University Hospital’s institutional review board reviewed and approved this study (CNUH-2018-210) before the study began, and written informed consent for each procedure was obtained from all participants included in the study.

### Polysomnography

Patients underwent an in-laboratory overnight level 1 PSG utilizing the Embla N7000 and RemLogic 2.0.0 (Embla Frameworks, Denver, CO, USA) programs or the NOX A1 (Nox Therapeutic, Reykjavík, Iceland) and Noxturnal programs. Standard PSG equipment was used: electrocardiography, electroencephalography, bilateral electrooculography, electromyography, pulse oximetry, oral thermistor, nasal pressure, dual degree thoracoabdominal respiratory inductance plethysmography belts, microphone, and piezoelectric position sensor. All data were recorded on a computerized sleep scoring system and scored according to the American Academy of Sleep Medicine Scoring Manual Form 2.5 [16]. AHI, lowest oxygen saturation during sleep, percentage of total sleep time with oxygen saturation < 90% (T90SaO_2_ < 90%), and sleep time spent in each position (supine or lateral) were determined. In addition, the position dependency of OSA was evaluated [17]. Positional obstructive sleep apnea (POSA) was defined as an AHI in the supine position that was at least twice that in the non-supine position [18]. Patients who slept for less than 20 min in either the supine or the non-supine sleep position were excluded from the positional dependence categorization [19].

### Drug-induced sleep endoscopy protocol

A multimodality DISE system was used for DISE procedures [20]. The procedure was performed according to the guidelines of the European position paper [7]. Briefly, DISE was performed in a quiet, light-dimmed operating room. Intravenous propofol was administered as the sedative agent. Vital signs and electrocardiography parameters were monitored. Bispectral index values were targeted at a sedation level of 60−70 [7]. The upper airway was evaluated according to the VOTE classification system at four different levels of the collapsible segment: the velum (palate), oropharyngeal lateral walls, tongue base, and epiglottis [21]. The velum, oropharyngeal lateral wall, and tongue structures were scored according to the degree of collapsibility: 0 = no obstruction (collapse less than 50%), 1 = partial (collapse between 50−75%), or 2 = complete (collapse >75%). The epiglottis was scored differently according to previous studies: 0 = no obstruction (collapse less than 50%), 1 = partial (collapse between <100%), or 2 = complete (collapse = 100%) [22, 23].

### Head rotation maneuvers during drug induced sleep endoscopy

There is no information on how much the head should be rotated during sleep, as described above. While previous studies suggest that the head can be rotated up to 60° during natural sleep [9, 10, 13], keeping the head at that angle might be difficult for many patients. Thus, we wanted to determine whether less rotation also positively influenced OSA, and thus, we analyzed the effect of head rotation at 30° as well as 60°. For the analysis, we performed DISE in the supine position, and we rotated the head of the patients to the right 30° (HRM30°) and 60° (HRM60°). Each position was observed for 1 min with the bispectral index level between 60 and 70, owing to the limited time available for the procedure. The range of head rotation was examined before sedation for safety. The effect of HRM was evaluated in terms of anatomic resolution of airway obstruction. A resolution was defined as a score of zero or one for all four sites (velum, oropharyngeal lateral walls, tongue base, and epiglottis).

### Statistical analysis

Statistical analyses were performed using SPSS software (IBM SPSS Statistics for Windows, Version 25.0. Armonk, NY: IBM Corp.). In Table 1, the normally distributed variables are summarized as mean and standard deviation (S.D.), and the non-normally distributed variables are summarized as the mean and interquartile ratio (IQR). In Table 2, McNemar’s test was used to compare the airway status between the supine position, HRM30°, and HRM60°. In Table 3, the Mann-Whitney U test or t-test was performed to compare the baseline characteristics between responders and non-responders to the HRM. We also performed multiple logistic regression to identify factors related to the responder group for AHI, weight, POSA, and obstruction sites (velum, tongue base, oropharyngeal lateral walls, and epiglottis).

**Table 1:** Pretreatment clinical characteristics of the study population*.

	Variable	Median (IQR)
Demographic factors	Age	52 (25–61)
	Sex (M/F)	175/46
	BMI (kg/m^2^)	26.7 (24.6–29.4)
PSG parameters	T90SaO2 < 90	5.5 (3.8–10.5)
	AHI (events/hour)	28.2 (13.7–71.9)
	LSAT (%)	82.7 (75.0–88.0)
	SUP_AHI (events/hour)	36.2 (16.0–66.2)
	Nsup_AHI (events/hour)	11.0 (3.0–27.2)
	POSA	91 (41.2%)
	Non-POSA	76 (34.4%)
DISE findings	No obstruction	18 (8.1%)
	Single-site obstruction	119 (53.8%)
	Multi-site obstruction	84 (38.0%)

*Non-POSA, patients without positional OSA; POSA, *positional OSA*, *M*, male; *F*, female; *BMI*, body mass index; *T90SaO*_***2*,**_ percentage of total sleep time with saturation <90%; *AHI*, apnea-hypopnea index; *LSAT*, lowest oxygen saturation during sleep; *SUP_AHI*, supine AHI; *Nsup_AHI*, non-supine AHI; DISE, drug induced sleep endoscopy. Data are expressed as the median (interquartile range).

^†^ Sites with a VOTE score of 2 were considered as an obstruction. Sigle-site obstruction; patients with a single site of the collapse, Multi-site obstruction; patients with multiple sites of collapse.

**Table 2:** Number of patients with airway obstruction according to the degrees of head rotation*.

	Patients with airway collapse (n)			The improvement according to head position (n, %) ^†^		
	0°	30°	60°	0°-30°	0°-60°	30°-60°
Any of the four sites	203	174	161	29 (14.3%)	42 (20.7%)	13 (7.5%)
				*(p < 0*.*001)*	(p < 0.001)	(p < 0.015)
Velum	188	164	150	24 (12.8%)	38 (20.2%)	14 (8.5%)
				(p < 0.001)	(p < 0.001)	(p < 0.001)
Oph lat wall	28	22	20	6 (21.4%)	8 (28.6%)	2 (9.1%)
				(p = 0.014)	(p = 0.005)	(p = 0.157)
Tongue base	54	42	33	12 (23.6%)	21 (40.0%)	9 (21.4%)
				(p < 0.001)	(p < 0.001)	(p = 0.003)
Epiglottis	38	24	18	14 (36.8%)	20 (52.6%)	6 (25.0%)
				(p < 0.001)	(p < 0.001)	(p = 0.014)

*Airway structures with a VOTE score of 2 during DISE were considered obstruction. Oph lat wall; Oropharyngeal lateral wall, 0°-30°, the number of responders with 30° head rotation from 0°; 0°-60°, the number of the responder in 60° head rotation from 0°; 30°-60°, the number of the responder in 60° head rotation from 30°.

†McNemar’s test was performed to analyze the effect of head rotation.

**Table 3:** Comparison of baseline characteristics between responders and non-responders to head rotation maneuver*.

		NON-RESPONDER	RESPONDER	*P*-VALUE^†^
Demographics	Number of patients	161	60	
	Age	52 (33–61)	52 (41–60)	0.311
	Sex (M/F)	127/34	48/12	1.000
	Weight (kg)	78.0 (67.7–87.0)	72.5 (66.0–80.8)	0.035
	Height (cm)	169.8 (162.0–173.7)	169.0 (161.1–171.8)	0.301
	BMI (kg/m2)	27.0 (24.7–30.0)	26.3 (24.2–28.3)	0.111
PSG parameters	T90SaO_2_ < 90	6.1 (3.9–12.1)	4.6 (3.5–6.6)	0.048
	AHI	34.6 (15.8–55.00)	18.4 (8.8–31.0)	< 0.001
	LSAT	81.7 (73.3–87.2)	83.8 (77.6–90.1)	0.130
	SUP_AHI	45.3 (18.8–69.4)	23.7 (10.9–45.3)	0.001
	Nsup_AHI	11.9 (2.4–29.0)	8.6 (3.5–20.9)	0.006
	POSA^‡^	55	36	0.002
	Non-POSA	62	14	
The site of	Velum	136 (84.5%)	52 (86.7%)	0.833
airway collapse	Oropharyngeal	27 (16.8%)	1 (1.7%)	0.001
on Supine DISE (0°)	Tongue base	39 (24.2%)	15(9.3%)	0.558
(n, %)	Epiglottis	26 (16.1%)	12 (7.5%)	0.549

* M, male; F, female; PSG, polysomnography; T90SaO2 < 90%, percentage of total sleep time with saturation < 90%; AHI, apnea-hypopnea index; LSAT, lowest oxygen saturation during sleep; SUP_AHI, supine AHI; Nsup_AHI, non-supine AHI; Non-POSA, patients without positional OSA; POSA, patients with positional OSA; Obs, obstruction; no-Obs, no obstruction. Data are expressed as the median (interquartile range).

^†^ The Mann-Whitney U test was used to analyze non-normally distributed variables. Fisher’s exact test was performed to analyze the sex distribution and obstruction sites.

^‡^ POSA was defined when a patient had an AHI in the supine position at least twice as high as that in the non-supine position. Patients who slept for less than 20 min in either the supine or non-supine sleep position were excluded from the categorization to avoid including the patients who slept for only a few minutes in either position to be wrongly classified as POSA patients.

## Results

It was postulated that if relatively limited head rotation successfully alleviated airway collapse, then it could be used as a potential therapy since a device to accomplish 30° seems quite feasible. Thus, we analyzed the airway with the head rotated to 0°, 30°, and 60°. We also identified the clinical characteristics of responders to head rotation.

In total, 243 patients were enrolled in the study (Fig 1). Twenty-two patients were excluded from the study: 6 refused PSG, 5 refused DISE, and 11 had incomplete examinations. Finally, a total of 221 patients were analyzed (Table 1). The median age was 52(25–61) years, with a body mass index of 26.7(24.6–29.4) kg/m^2^. The patients had a median AHI of 28.2 (13.7–71.9) events/h with minimum oxygen saturation of 82.7 (75.0–88.0)%. The supine AHI was 36.2 (16.0–66.2) events/h, and the non-supine AHI was 11.0 (3.0–27.2) events/h. Seventy-six patients were categorized as non-POSA, and 91 were categorized as POSA. Fifty-four patients did not fulfill the criteria for positional dependency (>20 min in both supine and lateral positions) and thus were not categorized [19].

### DISE findings and the effect of the degree of head rotation (HRM0° vs. HRM30° vs. HRM60°)

We analyzed whether limited head rotation had an effect on airway collapse in OSA patients. To do this, airway status in the HRM0°, HRM 30°, and HRM 60° were compared (Table 2). In HRM0°, 203 out of 221 patients (91.9%) had at least one anatomic site labeled as a 2, i.e., complete obstruction (Table 2). 84 patients (38.0%) had multi-site obstruction, and 119 (53.8%) had a single-site obstruction (Table 1). When we rotated the head to 30°, the collapsibility score changed from 2 to 0 in 29 patients (14.3%, p < 0.001). A further rotation to 60° resolved collapse in and additional 13 patients (7.5%, p < 0.015). These data suggest that relatively limited head rotation of 30° improves airway patency significantly (p < 0.001).

When we analyzed the DISE data according to the site of obstruction (Table 2), Velum collapse was resolved in 24 patients (12.8%, p<0.001) with HRM30°. HRM60° resolved velum collapse in an additional 14 patients (8.5%, p<0.001). Oropharyngeal lateral walls collapse was resolved in six patients (21.4%, p = 0.014) with HRM30° and an additional two (9.1%, p = 0.157) with HRM60°. Tongue base collapses resolved in 12 patients (23.6%) with HRM30° and an additional nine with HRM60°. Moreover, epiglottic collapse resolved in 14 patients (36.8%) with HRM 30° and an additional six with HRM60°. Due to multiple comparisons, a *p*-value <0.004 was considered significant because there are three head positions (0°, 30°, and 60°) for four anatomic sites (V, O, T, and E). With Bonferroni correction, the oropharyngeal lateral walls did not benefit from head rotation (p>0.004) even in HRM60°.

### Clinical characteristics of the responder group

We analyzed the responder group’s clinical characteristics (Table 3). A responder is a patient in whom all sites of airway obstruction resolved (VOTE 2 obstruction) with HRM. Thus, patients without airway obstruction at 0 degrees were excluded from the analysis. In univariate analysis (Table 3), age (p = 0.311) and sex (p = 1.000) did not affect the results. Responders had lower body weight (p<0.035), lower AHI (p<0.001), and positional OSA (p = 0.002). Notably, the patients with oropharyngeal lateral walls collapse responded significantly worse than others (p<0.001).

We also performed multivariate logistic regression analysis for the predictors AHI, weight, POSA, and obstruction sites (velum, tongue base, oropharyngeal lateral walls, and epiglottis). Lower AHI (*p* = 0.000, Exp(B): 0.961, 0.941−0.981) and absence of oropharyngeal lateral wall collapse (*p* = 0.011, Exp(B): 15.197, 1.89−122.43) were significant predictors of response.

## Discussion

The results from this experiment suggest that head rotation could be a potential therapeutic modality for patients with OSA since head rotation significantly improved the airway status of many patients. Not only did full rotation (60°) open the airway, but so did limited rotation (30°), which is likely easier to maintain during natural sleep. In addition, our data suggest that head rotation maneuvers are most effective in patients with less severe OSA and without oropharyngeal lateral wall collapse. These results inform how much the head should be rotated and to whom it should be applied. They may also enable the development of sleep aids targeting head rotation.

Our data showed that the HRM60° completely resolved the obstruction in the velum in 20.2% (p < 0.001), the tongue base in 40.0% (p < 0.001), and the epiglottic in 52.6% (p < 0.001) of patients. Again, “resolution” means the collapsibility score for that particular site of collapse changed from a 2 to a 0 or 1. Thus, head rotation improves airway obstruction in patients with OSA. The previous literature supports this result. Safiruddin et al. reported that the frequency of complete airway collapse decreased with head rotation compared to no head rotation, especially in the velum (32.8%, p < 0.001), tongue base (81.2%, p < 0.001), and epiglottic collapse (69.5%, p < 0.001) [7, 8]. Van Kesteren et al. and Tate et al. also reported on the positive influence of the head rotation on OSA severity in their observation during overnight PSG, as was mentioned in the introduction [7, 8].

However, it is not easy to maintain the head rotated fully, such as at 60°, and there are studies with only dichotomous criteria: rotated or not rotated [9–11, 13]. Thus, we tested whether less head rotation, which can be more easily achieved during sleep, also improves airway obstruction. We simulated 0°, 30°, and 60° rotations. We found that airway obstruction was significantly improved with 30° head rotation (p < 0.001, Table 2), especially in the velum, tongue base, and epiglottis.

We also analyzed the responder characteristics. The responder group tended to have lower AHI and an absence of oropharyngeal lateral wall collapse. Lower AHI is also a responder characteristic of positional therapy (lateral head and trunk position), which is known to have a similar anatomic effect on the airway as head rotation [12]. This finding is supported by a European Respiratory Society task force group, which stated that responders to positional therapy tend to have lower AHI [15]. In addition, the DISE finding in previous literature showed similar results that oropharyngeal lateral walls collapse would not respond well to head roll maneuver. A previous study in 637 patients revealed that only 17.8% of oropharyngeal lateral walls collapse responded to head rotation. Patients with oropharyngeal lateral walls collapse responded significantly worse than those with velum (27.1%. p<0.05), tongue base (60.6%, <0.001), and epiglottic (62.8%, p<0.001) collapse [24]. Furthermore, Safirrudin et al.’s study reported that oropharyngeal lateral wall collapse did not respond well to head rotation. In that study, only two out of seven oropharyngeal lateral wall obstructions responded to head rotation (p = 0.462) [11].

While previous studies suggest that head rotation reduces OSA severity, who would benefit the most or how to prescribe it was not clarified. Thus, this study focused on whether a relatively limited, feasible range of head rotation (30°) positively influences airway obstruction and who can benefit from head rotation. We found that a maintainable range of rotation, such as 30°, works effectively in many patients. In addition, patients with good response tended to have less severe OSA and collapse at sites other than the oropharyngeal lateral walls. With these results, we can determine and how and to whom to prescribe head rotation. This study suggests that head rotation could be used as a type of positional therapy for OSA, and it could encourage the development of sleep aids targeting head rotation.

However, this study is not without limitations. The study results were based on drug induced sleep findings, not natural sleep findings. Thus, there is a possibility that the findings of this study can be different from natural sleep. However, this study focused on airway status. A recent study from Park et al. reported that obstruction patterns of the airway appeared to be in agreement between DISE and natural sleep endoscopy [25]. In addition, the DISE study had some advantages over the natural sleep study. We can simulate our desired position and precisely control the patient’s position during DISE. However, we cannot control head position in natural sleep and it causes difficulty in the study. For example, Kerenstern et al. observed that people slept with the head rotated only 1.6% of the time in natural sleep [9]. Furthermore, natural sleep studies cannot rule out the effect of concurrent situations that can affect OSA severity, such as head flexion. In Tate’s study, only four patients (14.3%) slept with the head in the lateral position without head flexion or extension. Others did not sleep with their head in the lateral position at all, or the head lateral position was mixed with head flexion or extension [10]. Head position can be correlated with postural balance impairment in OSA patients, as shown in Demir et al.’s report [26]. Thus, The DISE study could exclude the confounding effect. In addition, positional sensors to determine the head and trunk position was not needed in the DISE study.

Another limitation is that we analyzed the DISE with the VOTE classification. The pharynx can be divided into 3 parts: nasopharynx, oropharynx, and hypopharynx. However, VOTE classification does not evaluate pharyngeal segments separately. Thus, using the nose, oropharynx, hypopharynx, and larynx (NOHL) criteria or other criteria that assess pharyngeal segments separately, may give us more detailed information. However, the VOTE criteria have strength in their comprehensiveness [27].

With this simulation of head rotation, we identified the influence of head rotation on patients with OSA according to the degree of rotation. In addition, we are aware of the clinical characteristics of patients who had an good response to the head rotation maneuver. Thus, with the development of a head position monitoring system that can quickly and accurately convert raw accelerometer data into the desired head position tilting angles and can easily be hooked up with PSG [28], the head rotation maneuver can be a potential therapeutic modality for patients with OSA and encourage the development of sleep gadgets to maintain head rotation during sleep.

## Conclusion

Head rotation improved airway patency in OSA patients, even with a relatively limited degree of head rotation. In addition, the responders to head rotation tended to have less severe AHI and no oropharyngeal lateral wall collapse. Therefore, if patients are adequately selected, the head rotation could be considered a potential therapeutic target for OSA.

## Supporting information

S1 Fig Comparison of airway responses to head rotation according to the sites. The epiglottis responses better than velum in supine to 30°, supine to 60° and 30° to 60° rotations. In addition, the tongue base also has a better response than the velum in supine to 60° rotation. V, Velum; O, Oropharyngeal lateral walls; T, Tongue base; E, Epiglottis; Sup to 30, Supine to 30°; Sup to 60, Supine to 60°; 30 to 60, 30° to 60°. P-values of less than 0.001 are denoted as ***, and less than 0.05 denotes *. A resolution of VOTE score 2 obstruction was considered as a response to the treatment.
(DOCX)

S2 Fig The comparison between the improvement of the site of obstruction according to non-POSA and POSA groups. The POSA group showed significant improvement in supine to 30° and supine to 60° for tongue base compared to the non-POSA group. V, Velum; O, Oropharyngeal lateral walls; T, Tongue base; E, Epiglottis; Sup to 30, Supine to 30° head rotation; Sup to 60, Supine to 60° head rotation; 30 to 60, 30° to 60° head rotation. P-values of less than 0.05 denote as *.
(DOCX)

S1 File (DOCX)
