# Supplementary material for: Head rotation improves airway obstruction, especially in patients with less severe obstructive sleep apnea without oropharyngeal collapse
Source: PLoS One. 2022 May 24;17(5):e0268455. doi: 10.1371/journal.pone.0268455 (PMC9129012; doi:10.1371/journal.pone.0268455)
Supplement: S1 Fig — The epiglottis responses better than velum in supine to 30°, supine to 60° and 30° to 60° rotations. In addition, the tongue base also has a better response than the velum in supine to 60° rotation. V, Velum; O, Oropharyngeal lateral walls; T, Tongue base; E, Epiglottis; Sup to 30, Supine to 30°; Sup to 60, Supine to 60°; 30 to 60, 30° to 60°. P-values of less than 0.001 are denoted as ***, and less than 0.05 denotes *. A resolution of VOTE score 2 obstruction was considered as a response to the treatment. (DOCX) [file pone.0268455.s001.docx]

###
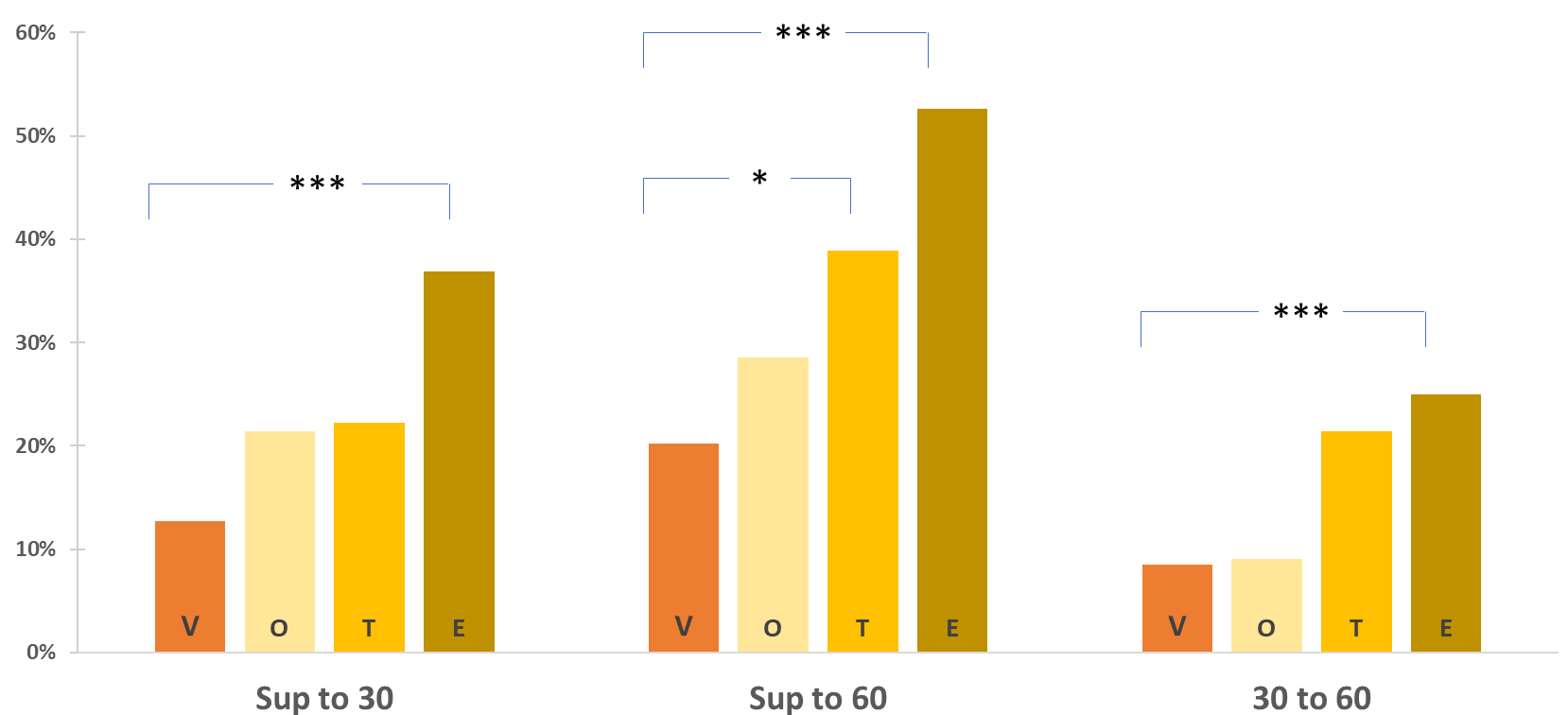
S1 Fig. Comparison of airway responses to head rotation according to the sites. The epiglottis responses better than velum in supine to 30°, supine to 60° and 30° to 60° rotations. In addition, the tongue base also has a better response than the velum in supine to 60° rotation. V, Velum; O, Oropharyngeal lateral walls; T, Tongue base; E, Epiglottis; Sup to 30, Supine to 30°; Sup to 60, Supine to 60°; 30 to 60, 30° to 60°. P-values of less than 0.001 are denoted as ***, and less than 0.05 denotes *. A resolution of VOTE score 2 obstruction was considered as a response to the treatment.
