# Supplementary material for: Head rotation improves airway obstruction, especially in patients with less severe obstructive sleep apnea without oropharyngeal collapse
Source: PLoS One. 2022 May 24;17(5):e0268455. doi: 10.1371/journal.pone.0268455 (PMC9129012; doi:10.1371/journal.pone.0268455)
Supplement: S2 Fig — The POSA group showed significant improvement in supine to 30° and supine to 60° for tongue base compared to the non-POSA group. V, Velum; O, Oropharyngeal lateral walls; T, Tongue base; E, Epiglottis; Sup to 30, Supine to 30° head rotation; Sup to 60, Supine to 60° head rotation; 30 to 60, 30° to 60° head rotation. P-values of less than 0.05 denote as *. (DOCX) [file pone.0268455.s002.docx]

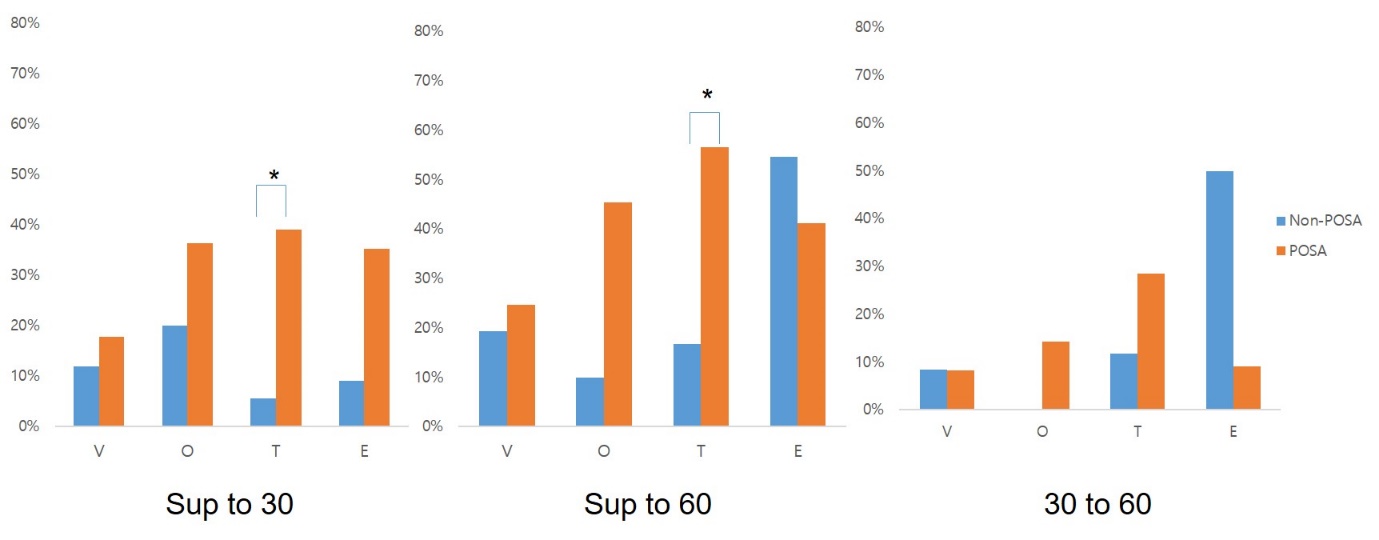


## S2 Fig. The comparison between the improvement of the site of obstruction according to non-POSA and POSA groups. The POSA group showed significant improvement in supine to 30° and supine to 60° for tongue base compared to the non-POSA group. V, Velum; O, Oropharyngeal lateral walls; T, Tongue base; E, Epiglottis; Sup to 30, Supine to 30° head rotation; Sup to 60, Supine to 60° head rotation; 30 to 60, 30° to 60° head rotation. P-values of less than 0.05 denote as *
