# Supplementary material for: Head rotation improves airway obstruction, especially in patients with less severe obstructive sleep apnea without oropharyngeal collapse
Source: PLoS One. 2022 May 24;17(5):e0268455. doi: 10.1371/journal.pone.0268455 (PMC9129012; doi:10.1371/journal.pone.0268455)
Supplement: S1 File — (DOCX) [file pone.0268455.s003.docx]

### Comparison of airway responses to head rotation according to the sites is demonstrated in S1 Fig. The epiglottis responds better than the velum in supine to 30°, supine to 60° and 30° to 60° rotations. In addition, the tongue base also has a better response than the velum in supine to 60° rotation. In addition, comparison between the improvement of the site of obstruction according to non-POSA and POSA groups (S2 Fig) showed that the POSA group had significant improvement in supine to 30° and supine to 60° for tongue base compared to the non-POSA group. V, Velum; O, Oropharyngeal lateral walls; T, Tongue base; E, Epiglottis; Sup to 30, Supine to 30° head rotation; Sup to 60, Supine to 60° head rotation; 30 to 60, 30° to 60° head rotation. P-values < 0.05 are indicated by *, a resolution of VOTE score 2 obstruction was considered as a response to the treatment.
