# Supplementary material for: Head rotation improves airway obstruction, especially in patients with less severe obstructive sleep apnea without oropharyngeal collapse
Source: PLoS One. 2022 May 24;17(5):e0268455. doi: 10.1371/journal.pone.0268455 (PMC9129012; doi:10.1371/journal.pone.0268455)
Supplement: Attachment — Submitted filename: Response to Reviewers 0323.docx [file pone.0268455.s004.docx]

**Response to Reviewers**

**JOURNAL REVISION LETTER**

March 6, 2022

Dear Prof. Joerg Heber, M.D.

We wish to submit a revised version of manuscript ID Ms. No. PONE-D-22-04206 entitled “Head rotation improves airway obstruction, especially in patients with less severe obstructive sleep apnea without oropharyngeal collapse” to be considered for publication in *PLOS One.*

Thank you for allowing us to revise and resubmit this manuscript. We appreciate the time and comments provided by you and each reviewer. We have incorporated the suggested changes into the revised manuscript to the best of our ability. The manuscript has certainly benefited from these insightful comments and suggestions. We look forward to working with you and the reviewers to move this manuscript closer to publication in *PLOS One.*

We have responded specifically to the comments and suggestions, as shown below. To make the changes easier to identify, the newly added changes have been numbered and additional text in the manuscript has been highlighted.

**Reviewer #1**

**Reviewer #1: The paper is interesting and well written. Minor corrections to improve the overall quality
Introduction**

- We thank the reviewer for their valuable time and recommendations.

1. **INTRODUCTION, line 2,**

**“among OSA treatment after a after adequate phenotyping with DISE, treatment with barbed pharyngoplasty sutures in lateral or anteroposterior collapse has shown excellent results. please cite doi:10.3390/ijerph17051542 and doi:10.1016/j.amjoto.2021.103197**

- We thank the reviewer for this valuable comment. We agree with your opinion. Therefore, we cited them as reference numbers 5 and 6.

***Added reference***

Reference #5: Iannella G, Lechien JR, Perrone T, Meccariello G, Cammaroto G, Cannavicci A, et al. Barbed reposition pharyngoplasty (BRP) in obstructive sleep apnea treatment: State of the art. Am J Otolaryngol. 2022;43(1):103197. Epub 2021/09/08. doi: 10.1016/j.amjoto.2021.103197. PubMed PMID: 34492427.

Reference #6: Iannella G, Vallicelli B, Magliulo G, Cammaroto G, Meccariello G, De Vito A, et al. Long-Term Subjective Outcomes of Barbed Reposition Pharyngoplasty for Obstructive Sleep Apnea Syndrome Treatment. Int J Environ Res Public Health. 2020;17(5). Epub 2020/03/04. doi: 10.3390/ijerph17051542. PubMed PMID: 32121007; PubMed Central PMCID: PMCPMC7084807.

2. **After ''Another problem with head rotation'' an interesting paper described an accelerometer-based sensing system for accurate head position monitoring is developed and realized. The core CORDIC- (COordinate Rotation DIgital Computer-) based tilting sensing algorithm is realized in the system to quickly and accurately convert accelerometer raw data into the desired head position tilting angles. The system can hook up with PSG devices for diagnosis to have head position information integrated with other PSG-monitored signals. please cite doi:10.1155/2017/4728187.**

- We thank the reviewer for sharing this article with us. It is indeed an interesting study and gave us some valuable insight; thus, we added this added the text and reference in the discussion.

***Added text in discussion***

“With this simulation of head rotation, we identified the influence of head rotation on patients with OSA according to the degree of rotation. In addition, we are aware of the clinical characteristics of patients who had a good response to the head rotation maneuver. Thus, with the development of a head position monitoring system that can quickly and accurately convert raw accelerometer data into the desired head position tilting angles and can easily be hooked up with PSG [29], the head rotation maneuver can be a potential therapeutic modality for patients with OSA and encourage the development of sleep gadgets to maintain head rotation during sleep.”

***Added reference***

Reference #29:	Lin WY, Chou WC, Shiao TH, Shiao GM, Luo CS, Lee MY. Realization of a CORDIC-Based Plug-In Accelerometer Module for PSG System in Head Position Monitoring for OSAS Patients. J Healthc Eng. 2017;2017:4728187. Epub 20170820. doi: 10.1155/2017/4728187. PubMed PMID: 29065608; PubMed Central PMCID: PMCPMC5585547.

3. **Methods: modify the flow chart in consort model 2010**

-> We thank the reviewer for this suggestion. Accordingly, we modified the flow chart as per the CONSORT 2010 Flow Diagram.

4. **Methods: fig. 1 patients were divided....cite the aasm 2017 guidelines**

- We thank the reviewer for this comment. According to your suggestion, we cited the 2017 AASM guidelines by Kapur et al.

Reference #17: Kapur VK, Auckley DH, Chowdhuri S, Kuhlmann DC, Mehra R, Ramar K, et al. Clinical Practice Guideline for Diagnostic Testing for Adult Obstructive Sleep Apnea: An American Academy of Sleep Medicine Clinical Practice Guideline. J Clin Sleep Med. 2017;13(3):479-504. Epub 20170315. doi: 10.5664/jcsm.6506. PubMed PMID: 28162150; PubMed Central PMCID: PMCPMC5337595.

5. **Method: which type of psg was performed? at home?**

- We thank the reviewer for your valuable comment. PSG was performed in the sleep lab at the hospital. We clarified this fact in the Materials and Methods section.

***Corrected text:***

“Patients underwent **an in-laboratory overnight level 1 PSG** utilizing the Embla N7000 and RemLogic 2.0.0 (Embla Frameworks, Denver, CO, USA) programs or the NOX A1 (Nox Therapeutic, Reykjavík, Iceland) and Noxturnal programs.”

6. **Discussion: Patients with obstructive sleep apnoea syndrome (OSAS) can be more prone to accidents due to excessive daytime sleepiness which can lead to attention deficits and thereby cause balance problems. One of the tests evaluating postural balance is static posturography (SPG). In this study, the authors evaluated postural balance with PSG in OSAS patients. Patients who were referred to a sleep disorders outpatient clinic of a tertiary health care centre with snoring, daytime sleepiness or witnessed apnoea were enrolled consecutively in this cross-sectional study. They were grouped as the OSAS group and the control group according to the apnoea-hypopnoea index. Posturographic analyses were carried out in all subjects on a SPG platform under five different conditions: eyes open (EO), eyes closed (EC), head rotated to left (HL), head rotated to right (HR), and tandem Romberg. A total of 95 patients and 23 controls were included in this study. In EO conditions, there was no difference between the OSAS group and the control group in any of the posturographic parameters. In EC conditions, change in lateral sway was significantly higher in the OSAS group which also correlated negatively with SaO2(min). HR conditions caused an increase in anterior-posterior (A-P) sway velocity, and HL conditions led to an increase in change in lateral and A-P sways, sway area, and sway area velocity in the OSAS group. Their findings suggested that postural balance in OSAS patients is impaired even in the very first hours of the day, and that the severity of the disease has an impact on postural balance. please cite doi:10.5603/PJNNS.a2019.0059.**

- We thank the reviewer for the valuable information. We agree with the reference you mentioned. Furthermore, we thought that postural imbalance can be correlated with the limitation of spontaneous non-maintain of head rotation in OSA patients, as shown in a previous study. Therefore, we added this reference according to your recommendation.

***Added reference***

Reference # 27: Demir T, Aslan K, Demirkiran M. Evaluation of postural balance in patients with obstructive sleep apnoea syndrome. Neurol Neurochir Pol. 2020;54(1):83-9. Epub 20191203. doi: 10.5603/PJNNS.a2019.0059. PubMed PMID: 31793660.

***Changed text***

However, this study is not without limitations. The study results were based on drug induced sleep findings, not natural sleep findings. Thus, there is a possibility that the findings of this study can be different from natural sleep. However, this study focused on airway status. A recent study from Park et al. reported that obstruction patterns of the airway appeared to be in agreement between DISE and natural sleep endoscopy [26]. In addition, the DISE study had some advantages over the natural sleep study. We can simulate our desired position and precisely control the patient's position during DISE. However, we cannot control head position in natural sleep and it causes difficulty in the study. For example, Kerenstern et al. observed that people slept with the head rotated only 1.6% of the time in natural sleep [9]. Furthermore, natural sleep studies cannot rule out the effect of concurrent situations that can affect OSA severity, such as head flexion. In Tate’s study, only four patients (14.3%) slept with the head in the lateral position without head flexion or extension. Others did not sleep with their head in the lateral position at all, or the head lateral position was mixed with head flexion or extension [10]. Head position can be correlated with postural balance impairment in OSA patients, as shown in Demir et al.’s report [27]. Thus, The DISE study could exclude the confounding effect. In addition, positional sensors to determine the head and trunk position was not needed in the DISE study.

**Reviewer #2**

1. **Response to Reviewer #2: discuss the difference between nohl and vote classification and if possible, apply in the paper. doi: 10.1007 / s00405-016-4081-7**

- We thank the reviewer for their time and comments. We agree that NOHL or other criteria that can differentiate the lateral pharyngeal wall into two or three segments will give us more information. Accordingly, we added this point to the limitation section.

**Added text**

Another limitation is that we analyzed the DISE with the VOTE classification. The pharynx can be divided into 3 parts: nasopharynx, oropharynx, and hypopharynx. However, VOTE classification does not evaluate pharyngeal segments separately. Thus, using the nose, oropharynx, hypopharynx, and larynx (NOHL) criteria or other criteria that assess pharyngeal segments separately, may give us more detailed information. However, the VOTE criteria have strength in their comprehensiveness [28].

2. **English editing for minor grammatical corrections.**

-> According to your recommendation, we submitted the revised manuscript for another round of English language editing.
