# Supplementary material for: Head rotation improves airway obstruction, especially in patients with less severe obstructive sleep apnea without oropharyngeal collapse
Source: PLoS One. 2022 May 24;17(5):e0268455. doi: 10.1371/journal.pone.0268455 (PMC9129012; doi:10.1371/journal.pone.0268455)
Supplement: Peer review history

# Peer review: Decision Letter 0

**DOI:** 10.1371/journal.pone.0268455.r001

**Authors:** Giovanni Cammaroto

21 Feb 2022

PONE-D-22-04206Head rotation improves airway obstruction, especially in patients with less severe obstructive sleep apnea without oropharyngeal collapsePLOS ONE

Dear Dr. Yang,

Thank you for submitting your manuscript to PLOS ONE. After careful consideration, we feel that it has merit but does not fully meet PLOS ONE’s publication criteria as it currently stands. Therefore, we invite you to submit a revised version of the manuscript that addresses the points raised during the review process.

Please submit your revised manuscript by Apr 07 2022 11:59PM. If you will need more time than this to complete your revisions, please reply to this message or contact the journal office at plosone@plos.org. When you're ready to submit your revision, log on to https://www.editorialmanager.com/pone/ and select the 'Submissions Needing Revision' folder to locate your manuscript file.

Please include the following items when submitting your revised manuscript:

- A rebuttal letter that responds to each point raised by the academic editor and reviewer(s). You should upload this letter as a separate file labeled 'Response to Reviewers'.
- A marked-up copy of your manuscript that highlights changes made to the original version. You should upload this as a separate file labeled 'Revised Manuscript with Track Changes'.
- An unmarked version of your revised paper without tracked changes. You should upload this as a separate file labeled 'Manuscript'.

If you would like to make changes to your financial disclosure, please include your updated statement in your cover letter. Guidelines for resubmitting your figure files are available below the reviewer comments at the end of this letter.

If applicable, we recommend that you deposit your laboratory protocols in protocols.io to enhance the reproducibility of your results. Protocols.io assigns your protocol its own identifier (DOI) so that it can be cited independently in the future. For instructions see: https://journals.plos.org/plosone/s/submission-guidelines#loc-laboratory-protocols. Additionally, PLOS ONE offers an option for publishing peer-reviewed Lab Protocol articles, which describe protocols hosted on protocols.io. Read more information on sharing protocols at https://plos.org/protocols?utm_medium=editorial-email&utm_source=authorletters&utm_campaign=protocols.

We look forward to receiving your revised manuscript.

Kind regards,

Giovanni Cammaroto

Academic Editor

PLOS ONE

Journal Requirements:

When submitting your revision, we need you to address these additional requirements.

1. Please ensure that your manuscript meets PLOS ONE's style requirements, including those for file naming. The PLOS ONE style templates can be found at 

https://journals.plos.org/plosone/s/file?id=wjVg/PLOSOne_formatting_sample_main_body.pdf and 

https://journals.plos.org/plosone/s/file?id=ba62/PLOSOne_formatting_sample_title_authors_affiliations.pdf

2. Thank you for stating the following in the Competing Interests section: 

"Conflicts of interest/Competing interests: Andrew Wellman works as a consultant for Apnimed, Nox, Inspire, and Somnifix. He has received grants from Regeneron and Somnifix. He also has a financial interest in Apnimed Corp., a company developing pharmacologic therapies for sleep apnea. Dr. Wellman’s interests were reviewed and are managed by Brigham and Women’s Hospital and Partners HealthCare in accordance with their conflict of interest policies. DPW is the Director of Medical Affairs for Apnimed and is a consultant for Cryosa, Cerebra Health, Philips Respironics, and LinguaFlex. "

Please confirm that this does not alter your adherence to all PLOS ONE policies on sharing data and materials, by including the following statement: "This does not alter our adherence to  PLOS ONE policies on sharing data and materials.” (as detailed online in our guide for authors http://journals.plos.org/plosone/s/competing-interests).  If there are restrictions on sharing of data and/or materials, please state these. Please note that we cannot proceed with consideration of your article until this information has been declared. 

Please include your updated Competing Interests statement in your cover letter; we will change the online submission form on your behalf.

3. Please review your reference list to ensure that it is complete and correct. If you have cited papers that have been retracted, please include the rationale for doing so in the manuscript text, or remove these references and replace them with relevant current references. Any changes to the reference list should be mentioned in the rebuttal letter that accompanies your revised manuscript. If you need to cite a retracted article, indicate the article’s retracted status in the References list and also include a citation and full reference for the retraction notice.

Additional Editor Comments:

Dear Authors,

I would like to thank you for your contribution. You will see that reviewers are asking for minor revisions

[Note: HTML markup is below. Please do not edit.]

Reviewers' comments:

Reviewer's Responses to Questions

**Comments to the Author**

1. Is the manuscript technically sound, and do the data support the conclusions?

The manuscript must describe a technically sound piece of scientific research with data that supports the conclusions. Experiments must have been conducted rigorously, with appropriate controls, replication, and sample sizes. The conclusions must be drawn appropriately based on the data presented. 

Reviewer #1: Yes

Reviewer #2: Yes

**********

2. Has the statistical analysis been performed appropriately and rigorously? 

Reviewer #1: Yes

Reviewer #2: Yes

**********

3. Have the authors made all data underlying the findings in their manuscript fully available?

The PLOS Data policy requires authors to make all data underlying the findings described in their manuscript fully available without restriction, with rare exception (please refer to the Data Availability Statement in the manuscript PDF file). The data should be provided as part of the manuscript or its supporting information, or deposited to a public repository. For example, in addition to summary statistics, the data points behind means, medians and variance measures should be available. If there are restrictions on publicly sharing data—e.g. participant privacy or use of data from a third party—those must be specified.

Reviewer #1: Yes

Reviewer #2: Yes

**********

4. Is the manuscript presented in an intelligible fashion and written in standard English?

PLOS ONE does not copyedit accepted manuscripts, so the language in submitted articles must be clear, correct, and unambiguous. Any typographical or grammatical errors should be corrected at revision, so please note any specific errors here.

Reviewer #1: Yes

Reviewer #2: Yes

**********

5. Review Comments to the Author

Please use the space provided to explain your answers to the questions above. You may also include additional comments for the author, including concerns about dual publication, research ethics, or publication ethics. (Please upload your review as an attachment if it exceeds 20,000 characters)

Reviewer #1: The paper is interesting and well written. Minor corrections to improve the overall quality

Introduction

- line 2, among OSA treatment after a after adequate phenotyping with DISE, treatment with barbed pharyngoplasty sutures in lateral or anteroposterior collapse has shown excellent results. please cite doi:10.3390/ijerph17051542 and doi:10.1016/j.amjoto.2021.103197

- after ''Another problem with head rotation'' an interesting paper described an accelerometer-based sensing system for accurate head position monitoring is developed and realized. The core CORDIC- (COordinate Rotation DIgital Computer-) based tilting sensing algorithm is realized in the system to quickly and accurately convert accelerometer raw data into the desired head position tilting angles. The system can hook up with PSG devices for diagnosis to have head position information integrated with other PSG-monitored signals. please cite doi:10.1155/2017/4728187.

Methods

- modify the flow chart in consort model 2010

- fig. 1 patients were divided....cite the aasm 2017 guidelines

- which type of psg was performed? at home?

Discussion

- Patients with obstructive sleep apnoea syndrome (OSAS) can be more prone to accidents due to excessive daytime sleepiness which can lead to attention deficits and thereby cause balance problems. One of the tests evaluating postural balance is static posturography (SPG). In this study, the authors evaluated postural balance with PSG in OSAS patients. Patients who were referred to a sleep disorders outpatient clinic of a tertiary health care centre with snoring, daytime sleepiness or witnessed apnoea were enrolled consecutively in this cross-sectional study. They were grouped as the OSAS group and the control group according to the apnoea-hypopnoea index. Posturographic analyses were carried out in all subjects on a SPG platform under five different conditions: eyes open (EO), eyes closed (EC), head rotated to left (HL), head rotated to right (HR), and tandem Romberg. A total of 95 patients and 23 controls were included in this study. In EO conditions, there was no difference between the OSAS group and the control group in any of the posturographic parameters. In EC conditions, change in lateral sway was significantly higher in the OSAS group which also correlated negatively with SaO2(min). HR conditions caused an i ncrease in anterior-posterior (A-P) sway velocity, and HL conditions led to an increase in change in lateral and A-P sways, sway area, and sway area velocity in the OSAS group. Their findings suggested that postural balance in OSAS patients is impaired even in the very first hours of the day, and that the severity of the disease has an impact on postural balance.

please cite doi:10.5603/PJNNS.a2019.0059.

Reviewer #2: The paper is interesting and only minor corrections should be made:

- discuss the difference between nohl and vote classification and if possible apply in the paper. doi: 10.1007 / s00405-016-4081-7

- english editing for minor grammatical corrections.

**********

6. PLOS authors have the option to publish the peer review history of their article (what does this mean?). If published, this will include your full peer review and any attached files.

If you choose “no”, your identity will remain anonymous but your review may still be made public.

**Do you want your identity to be public for this peer review?** For information about this choice, including consent withdrawal, please see our Privacy Policy.

Reviewer #1: No

Reviewer #2: No

[NOTE: If reviewer comments were submitted as an attachment file, they will be attached to this email and accessible via the submission site. Please log into your account, locate the manuscript record, and check for the action link "View Attachments". If this link does not appear, there are no attachment files.]

While revising your submission, please upload your figure files to the Preflight Analysis and Conversion Engine (PACE) digital diagnostic tool, https://pacev2.apexcovantage.com/. PACE helps ensure that figures meet PLOS requirements. To use PACE, you must first register as a user. Registration is free. Then, login and navigate to the UPLOAD tab, where you will find detailed instructions on how to use the tool. If you encounter any issues or have any questions when using PACE, please email PLOS at figures@plos.org. Please note that Supporting Information files do not need this step.

# Author comment: Author response to Decision Letter 0

**DOI:** 10.1371/journal.pone.0268455.r002

14 Apr 2022

Please refer to the attached file, named response to reviewers

March 6, 2022

Dear Prof. Joerg Heber, M.D.

We wish to submit a revised version of manuscript ID Ms. No. PONE-D-22-04206 entitled “Head rotation improves airway obstruction, especially in patients with less severe obstructive sleep apnea without oropharyngeal collapse” to be considered for publication in PLOS One.

Thank you for allowing us to revise and resubmit this manuscript. We appreciate the time and comments provided by you and each reviewer. We have incorporated the suggested changes into the revised manuscript to the best of our ability. The manuscript has certainly benefited from these insightful comments and suggestions. We look forward to working with you and the reviewers to move this manuscript closer to publication in PLOS One. 

We have responded specifically to the comments and suggestions, as shown below. To make the changes easier to identify, the newly added changes have been numbered and additional text in the manuscript has been highlighted.

 

Reviewer #1

Reviewer #1: The paper is interesting and well written. Minor corrections to improve the overall quality

Introduction

− We thank the reviewer for their valuable time and recommendations.

1. INTRODUCTION, line 2, 

“among OSA treatment after a after adequate phenotyping with DISE, treatment with barbed pharyngoplasty sutures in lateral or anteroposterior collapse has shown excellent results. please cite doi:10.3390/ijerph17051542 and doi:10.1016/j.amjoto.2021.103197

� We thank the reviewer for this valuable comment. We agree with your opinion. Therefore, we cited them as reference numbers 5 and 6.

Added reference

Reference #5: Iannella G, Lechien JR, Perrone T, Meccariello G, Cammaroto G, Cannavicci A, et al. Barbed reposition pharyngoplasty (BRP) in obstructive sleep apnea treatment: State of the art. Am J Otolaryngol. 2022;43(1):103197. Epub 2021/09/08. doi: 10.1016/j.amjoto.2021.103197. PubMed PMID: 34492427.

Reference #6: Iannella G, Vallicelli B, Magliulo G, Cammaroto G, Meccariello G, De Vito A, et al. Long-Term Subjective Outcomes of Barbed Reposition Pharyngoplasty for Obstructive Sleep Apnea Syndrome Treatment. Int J Environ Res Public Health. 2020;17(5). Epub 2020/03/04. doi: 10.3390/ijerph17051542. PubMed PMID: 32121007; PubMed Central PMCID: PMCPMC7084807.

2. After ''Another problem with head rotation'' an interesting paper described an accelerometer-based sensing system for accurate head position monitoring is developed and realized. The core CORDIC- (COordinate Rotation DIgital Computer-) based tilting sensing algorithm is realized in the system to quickly and accurately convert accelerometer raw data into the desired head position tilting angles. The system can hook up with PSG devices for diagnosis to have head position information integrated with other PSG-monitored signals. please cite doi:10.1155/2017/4728187.

� We thank the reviewer for sharing this article with us. It is indeed an interesting study and gave us some valuable insight; thus, we added this added the text and reference in the discussion.

Added text in discussion

“With this simulation of head rotation, we identified the influence of head rotation on patients with OSA according to the degree of rotation. In addition, we are aware of the clinical characteristics of patients who had a good response to the head rotation maneuver. Thus, with the development of a head position monitoring system that can quickly and accurately convert raw accelerometer data into the desired head position tilting angles and can easily be hooked up with PSG [29], the head rotation maneuver can be a potential therapeutic modality for patients with OSA and encourage the development of sleep gadgets to maintain head rotation during sleep.”

Added reference

Reference #29: Lin WY, Chou WC, Shiao TH, Shiao GM, Luo CS, Lee MY. Realization of a CORDIC-Based Plug-In Accelerometer Module for PSG System in Head Position Monitoring for OSAS Patients. J Healthc Eng. 2017;2017:4728187. Epub 20170820. doi: 10.1155/2017/4728187. PubMed PMID: 29065608; PubMed Central PMCID: PMCPMC5585547.

3. Methods: modify the flow chart in consort model 2010

-> We thank the reviewer for this suggestion. Accordingly, we modified the flow chart as per the CONSORT 2010 Flow Diagram.

4. Methods: fig. 1 patients were divided....cite the aasm 2017 guidelines

� We thank the reviewer for this comment. According to your suggestion, we cited the 2017 AASM guidelines by Kapur et al.

Reference #17: Kapur VK, Auckley DH, Chowdhuri S, Kuhlmann DC, Mehra R, Ramar K, et al. Clinical Practice Guideline for Diagnostic Testing for Adult Obstructive Sleep Apnea: An American Academy of Sleep Medicine Clinical Practice Guideline. J Clin Sleep Med. 2017;13(3):479-504. Epub 20170315. doi: 10.5664/jcsm.6506. PubMed PMID: 28162150; PubMed Central PMCID: PMCPMC5337595.

5. Method: which type of psg was performed? at home?

� We thank the reviewer for your valuable comment. PSG was performed in the sleep lab at the hospital. We clarified this fact in the Materials and Methods section.

Corrected text: 

“Patients underwent an in-laboratory overnight level 1 PSG utilizing the Embla N7000 and RemLogic 2.0.0 (Embla Frameworks, Denver, CO, USA) programs or the NOX A1 (Nox Therapeutic, Reykjavík, Iceland) and Noxturnal programs.”

6. Discussion: Patients with obstructive sleep apnoea syndrome (OSAS) can be more prone to accidents due to excessive daytime sleepiness which can lead to attention deficits and thereby cause balance problems. One of the tests evaluating postural balance is static posturography (SPG). In this study, the authors evaluated postural balance with PSG in OSAS patients. Patients who were referred to a sleep disorders outpatient clinic of a tertiary health care centre with snoring, daytime sleepiness or witnessed apnoea were enrolled consecutively in this cross-sectional study. They were grouped as the OSAS group and the control group according to the apnoea-hypopnoea index. Posturographic analyses were carried out in all subjects on a SPG platform under five different conditions: eyes open (EO), eyes closed (EC), head rotated to left (HL), head rotated to right (HR), and tandem Romberg. A total of 95 patients and 23 controls were included in this study. In EO conditions, there was no difference between the OSAS group and the control group in any of the posturographic parameters. In EC conditions, change in lateral sway was significantly higher in the OSAS group which also correlated negatively with SaO2(min). HR conditions caused an increase in anterior-posterior (A-P) sway velocity, and HL conditions led to an increase in change in lateral and A-P sways, sway area, and sway area velocity in the OSAS group. Their findings suggested that postural balance in OSAS patients is impaired even in the very first hours of the day, and that the severity of the disease has an impact on postural balance. please cite doi:10.5603/PJNNS.a2019.0059.

� We thank the reviewer for the valuable information. We agree with the reference you mentioned. Furthermore, we thought that postural imbalance can be correlated with the limitation of spontaneous non-maintain of head rotation in OSA patients, as shown in a previous study. Therefore, we added this reference according to your recommendation. 

Added reference

Reference # 27: Demir T, Aslan K, Demirkiran M. Evaluation of postural balance in patients with obstructive sleep apnoea syndrome. Neurol Neurochir Pol. 2020;54(1):83-9. Epub 20191203. doi: 10.5603/PJNNS.a2019.0059. PubMed PMID: 31793660.

Changed text

However, this study is not without limitations. The study results were based on drug induced sleep findings, not natural sleep findings. Thus, there is a possibility that the findings of this study can be different from natural sleep. However, this study focused on airway status. A recent study from Park et al. reported that obstruction patterns of the airway appeared to be in agreement between DISE and natural sleep endoscopy [26]. In addition, the DISE study had some advantages over the natural sleep study. We can simulate our desired position and precisely control the patient's position during DISE. However, we cannot control head position in natural sleep and it causes difficulty in the study. For example, Kerenstern et al. observed that people slept with the head rotated only 1.6% of the time in natural sleep [9]. Furthermore, natural sleep studies cannot rule out the effect of concurrent situations that can affect OSA severity, such as head flexion. In Tate’s study, only four patients (14.3%) slept with the head in the lateral position without head flexion or extension. Others did not sleep with their head in the lateral position at all, or the head lateral position was mixed with head flexion or extension [10]. Head position can be correlated with postural balance impairment in OSA patients, as shown in Demir et al.’s report [27]. Thus, The DISE study could exclude the confounding effect. In addition, positional sensors to determine the head and trunk position was not needed in the DISE study.

 

Reviewer #2

1. Response to Reviewer #2: discuss the difference between nohl and vote classification and if possible, apply in the paper. doi: 10.1007 / s00405-016-4081-7

� We thank the reviewer for their time and comments. We agree that NOHL or other criteria that can differentiate the lateral pharyngeal wall into two or three segments will give us more information. Accordingly, we added this point to the limitation section. 

Added text

Another limitation is that we analyzed the DISE with the VOTE classification. The pharynx can be divided into 3 parts: nasopharynx, oropharynx, and hypopharynx. However, VOTE classification does not evaluate pharyngeal segments separately. Thus, using the nose, oropharynx, hypopharynx, and larynx (NOHL) criteria or other criteria that assess pharyngeal segments separately, may give us more detailed information. However, the VOTE criteria have strength in their comprehensiveness [28].

2. English editing for minor grammatical corrections.

-> According to your recommendation, we submitted the revised manuscript for another round of English language editing.

Attachment Submitted filename: Response to Reviewers 0323.docx Click here for additional data file.

# Peer review: Decision Letter 1

**DOI:** 10.1371/journal.pone.0268455.r003

**Authors:** Giovanni Cammaroto

1 May 2022

Head rotation improves airway obstruction, especially in patients with less severe obstructive sleep apnea without oropharyngeal collapse

PONE-D-22-04206R1

Dear Dr. Yang,

We’re pleased to inform you that your manuscript has been judged scientifically suitable for publication and will be formally accepted for publication once it meets all outstanding technical requirements.

Within one week, you’ll receive an e-mail detailing the required amendments. When these have been addressed, you’ll receive a formal acceptance letter and your manuscript will be scheduled for publication.

An invoice for payment will follow shortly after the formal acceptance. To ensure an efficient process, please log into Editorial Manager at http://www.editorialmanager.com/pone/, click the 'Update My Information' link at the top of the page, and double check that your user information is up-to-date. If you have any billing related questions, please contact our Author Billing department directly at authorbilling@plos.org.

If your institution or institutions have a press office, please notify them about your upcoming paper to help maximize its impact. If they’ll be preparing press materials, please inform our press team as soon as possible -- no later than 48 hours after receiving the formal acceptance. Your manuscript will remain under strict press embargo until 2 pm Eastern Time on the date of publication. For more information, please contact onepress@plos.org.

Kind regards,

Giovanni Cammaroto

Academic Editor

PLOS ONE

Additional Editor Comments (optional):

Reviewers' comments:

Reviewer's Responses to Questions

**Comments to the Author**

1. If the authors have adequately addressed your comments raised in a previous round of review and you feel that this manuscript is now acceptable for publication, you may indicate that here to bypass the “Comments to the Author” section, enter your conflict of interest statement in the “Confidential to Editor” section, and submit your "Accept" recommendation.

Reviewer #1: All comments have been addressed

Reviewer #2: All comments have been addressed

**********

2. Is the manuscript technically sound, and do the data support the conclusions?

The manuscript must describe a technically sound piece of scientific research with data that supports the conclusions. Experiments must have been conducted rigorously, with appropriate controls, replication, and sample sizes. The conclusions must be drawn appropriately based on the data presented. 

Reviewer #1: Yes

Reviewer #2: Yes

**********

3. Has the statistical analysis been performed appropriately and rigorously? 

Reviewer #1: Yes

Reviewer #2: Yes

**********

4. Have the authors made all data underlying the findings in their manuscript fully available?

The PLOS Data policy requires authors to make all data underlying the findings described in their manuscript fully available without restriction, with rare exception (please refer to the Data Availability Statement in the manuscript PDF file). The data should be provided as part of the manuscript or its supporting information, or deposited to a public repository. For example, in addition to summary statistics, the data points behind means, medians and variance measures should be available. If there are restrictions on publicly sharing data—e.g. participant privacy or use of data from a third party—those must be specified.

Reviewer #1: Yes

Reviewer #2: Yes

**********

5. Is the manuscript presented in an intelligible fashion and written in standard English?

PLOS ONE does not copyedit accepted manuscripts, so the language in submitted articles must be clear, correct, and unambiguous. Any typographical or grammatical errors should be corrected at revision, so please note any specific errors here.

Reviewer #1: Yes

Reviewer #2: Yes

**********

6. Review Comments to the Author

Please use the space provided to explain your answers to the questions above. You may also include additional comments for the author, including concerns about dual publication, research ethics, or publication ethics. (Please upload your review as an attachment if it exceeds 20,000 characters)

Reviewer #1: INTERESTING STUDY ABOUT THE USE OF DISE TO EVALUATE EFFECT OF HEAD ROTATION IN POSA PATIENTS.

THE MANUSCRIPT HAS BEEN WELL IMPROVED AFTER REVISIONS

Reviewer #2: The authors performed all the changes required. the paper now is interesting and well structured. It's a pleasure to read the discussion. All the changes addressed now improved the quality of the manuscript. Well done!

**********

7. PLOS authors have the option to publish the peer review history of their article (what does this mean?). If published, this will include your full peer review and any attached files.

If you choose “no”, your identity will remain anonymous but your review may still be made public.

**Do you want your identity to be public for this peer review?** For information about this choice, including consent withdrawal, please see our Privacy Policy.

Reviewer #1: No

Reviewer #2: No

# Editor report: Acceptance letter

**DOI:** 10.1371/journal.pone.0268455.r004

**Authors:** Giovanni Cammaroto

12 May 2022

PONE-D-22-04206R1 

Head rotation improves airway obstruction, especially in patients with less severe obstructive sleep apnea without oropharyngeal collapse 

Dear Dr. Yang:

I'm pleased to inform you that your manuscript has been deemed suitable for publication in PLOS ONE. Congratulations! Your manuscript is now with our production department. 

If your institution or institutions have a press office, please let them know about your upcoming paper now to help maximize its impact. If they'll be preparing press materials, please inform our press team within the next 48 hours. Your manuscript will remain under strict press embargo until 2 pm Eastern Time on the date of publication. For more information please contact onepress@plos.org.

If we can help with anything else, please email us at plosone@plos.org. 

Thank you for submitting your work to PLOS ONE and supporting open access. 

Kind regards, 

PLOS ONE Editorial Office Staff

on behalf of

Dr. Giovanni Cammaroto 

Academic Editor

PLOS ONE
